# Dataset on the questionnaire-based survey of sharing services users’ motivation

**DOI:** 10.1016/j.dib.2020.106502

**Published:** 2020-11-05

**Authors:** Olga Saginova, Natalya Kireeva, Yury Saginov, Dmitry Zavyalov

**Affiliations:** Plekhanov Russian University of Economics, Entrepreneurship and Logistics Department, 117997, 36 Stremyanny, Moscow, Russia

**Keywords:** Sharing economy, Collaborative consumption, Sustainable development, Sharing services, Digital platforms, Intrinsic and extrinsic motivation, Ecological, Economic and social motives

## Abstract

The data set presents data collected by an online survey with a questionnaire using a Likert scale. The survey sample included 184 adults (18+), active and potential users of different sharing services platforms. The questionnaire is structured into six sections, including questions about respondents' attitudes to sharing services, frequency of usage and intentions to use these services in the future, and four groups of motives to the usage of sharing services platforms: enjoyment, reputation, sustainability, and economy sections. Each group of motives includes three to five statements with a 1–7 Likert scale to assess respondents' level of agreement or disagreement. The data table is supplemented by the original questionnaire in English and Russian. The data can be used to understand sharing services motivation to evaluate correlation with sustainable development triple base motives, formulate hypotheses, to draft a survey strategy and design, and to verify results, etc. The provided questionnaire is a ready-to-deploy instrument for customers surveys in the related areas of research.

## Specifications Table

**Table:** 

Subject	Management of Technology and innovationMarketing
Specific subject area	Managing and promoting the usage of sharing services platforms
Type of data	Table
How data were acquired	Online survey
Data format	Raw
Parameters for data collection	The survey sample included 184 adults (18 +) – active and potential users of sharing platforms services. The survey used snow-ball approach to sample construction when each of the initial group of respondents was asked to invite several people from different age and gender groups to fill in the online questionnaire
Description of data collection	The data were collected using online questionnaire available in two languages – Russian and English. Matters of interest included: – experience in sharing services usage – readiness to use sharing services – motives to use sharing services: • enjoyment and novelty • reputation among the community • sustainability sustainable consumption • economic motives
Data source location	Institution: Plekhanov Russian University of Economics City/Town/Region: Moscow Country: Russia
Data accessibility	Dataset on the questionnaire-based survey of sharing services users' motivation DOI:10.17632/c5k8wjrhd9.1 https://data.mendeley.com/datasets/c5k8wjrhd9/1 Questionnaires to the dataset on sharing ENG RUS DOI:10.17632/vzj6rd9crd.1 https://data.mendeley.com/datasets/vzj6rd9crd/1
Related research article	Saginov.Y., Zavyalov D., Saginova O. Distributed use economy: main concepts and characteristics // Russian Journal of Innovation Economics, Vol. 10, Number 3 (July – September 2020) DOI:10.18334/vinec.10.3.110726 (in Russian) [1]

## Value of the Data

• The data can be used to analyze individuals' attitudes towards sharing services platforms and their perceived benefits. The motivation groups were formed based on research publications, so these can be used in different countries and regions.
• The data's primary beneficiaries include sharing services researchers, digital platforms entrepreneurs, sociologists, and marketing specialists.
• The data can be used in further research to formulate the initial hypothesis, draft a survey strategy and design, and compare and verify results.
• The provided questionnaire is a ready-to-deploy instrument for similar surveys in any other city or for customer surveys in the related areas of research. The provided data can also serve as a valid control sample for proper verification.
• A deeper data analysis of the can be valuable for further research of motivations for the use of sharing services and their correlation to the sustainable development triple objectives. The importance of sustainable development values for the motivations in using sharing services can be estimated.

## 1 Data Description

This article is associated with a Microsoft Excel Worksheet as supplementary material. The data file contains respondents' agreements or disagreements with the “questionnaire” statements. Each statement has 184 entries of encoded answers. The respondents evaluated agreements and disagreements using a 7-point Likert scale, where 7 – for “completely agree” and 1 for “completely disagree.” Data interpretation comments are available in the questionnaire and/or the data file.

The original questionnaire is provided in two languages, Russian and English.

The questionnaire is anonymous; respondents' names were not included in the data to maintain privacy. Questions about motivations were tested for reliability by calculating the Cronbach alpha. All the results obtained were more than 0.7; therefore, there is a good internal consistency of the questionnaire's statement groups.

## 2 Experimental Design, Materials and Methods

The data were collected in May-June 2020 during the survey of sharing services users. The data were acquired using an online questionnaire.

The assessed characteristics of the sample included the following:

– sharing services users' share in the population and its growth potential,
– motivation of consumers for using sharing services platforms,
– sharing services consumers' intentions to use these services in the future,
– individuals' assessment of the sharing services' influence on sustainable development.

The structured questionnaire used for the data collection followed Malhorta's basic requirements and guidelines [2] to questionnaire design and contained the following sections:

1 Screening questions to filter and verify respondents' status:
  – age
  – gender
  – occupation
  – frequency of sharing services use
2 Main section including statements aimed at assessment of an individual's perception of sharing services motivations, including sub-section “attitude”, and sub-section “behavior” with four basic motivations: enjoyment, sustainability, reputation, and economy [3].

The survey sample included 184 adult citizens (18+) of Moscow – active and potential users of sharing services platforms who use or can use the sharing platforms such as cloud services, house-sharing, car-sharing, bike-sharing, coworking, coliving, etc. as of June 2020.

The survey used convenience sampling and “snowball” method of sample construction. Each representative of the target group, a group of people who use sharing services platforms, asked several people who belong to this group to participate in the online survey. The initial group of respondents was formed from graduate students of the Plekhanov Russian University of Economics. The structure of the resulting sample is shown in Table 1.

**Table 1:** The sample gender-age distribution

Age cohort	Women	Men	Total	Women,%	Men, %	Total, %
18–25 y. o.	57	41	98	58	42	100%
26–35 y. o.	21	11	32	65	35	100%
36–45 y. o.	22	1	23	96	4	100%
46–59 y. o.	9	10	19	47	53	100%
60 y. o. and older	5	3	8	62	38	100%
Total	114	70	184	62	38	100%

As seen from Table 1 the sample included five age cohorts: 18–25, 26–35, 36–45, 46–59, and 60 years and older. For each age cohort, the number of male and female respondents are given in columns 2 and 3, and the total number of respondents for each age cohort is presented in column 4. Columns 5 and 6 show the percentage of male and female respondents for each cohort. As can be expected, the number of respondents from cohorts of younger consumers (18–25 and 26–35 years old) makes the majority of respondents.

This “snowball” sampling approach has some limitations, such as a high probability of similar consumer preferences; individual respondents may know each other, and respondents may have an equivalent level of income. However, the information obtained can be used in exploratory research to study consumers' attitudes and motivations to the new business model for sharing consumption and perspectives for the development of the sharing services in the Russian Federation.

The questionnaire employed psychometric measurement [4]. We measured each construct with four or five items that were all on a 7-point Likert scale. For example, for the “Attitude” section, the following four statements were used:

– All things considered, I find participating in collaborative consumption to be a wise move.
– All things considered, I think participating in collaborative consumption is a good thing.
– Overall, sharing goods and services within a collaborative consumption community makes sense.
– Collaborative consumption is a better mode of consumption than selling and buying.

All items were adapted from existing prominent published sources [5,6].

## Ethics Statement

We agree upon standards of expected ethical behavior for all parties involved in the act of publishing. Our paper presents an accurate account of the work performed and an objective discussion of its significance. Underlying data is represented accurately in the article.

Each respondent was informed that his/her answers would be used as a part of a research project and agreed to that by filling in the questionnaire.

## CRediT Author Statement

Olga Saginova – conceptualization, methodology, writing, review, and editing

Natalya Kireeva – software, data curation

Yury Saginov – conceptualization, writing original draft

Dmitry Zavyalov – conceptualization, supervision, funding acquisition

## Declaration of Competing Interest

The authors declare that they have no known competing financial interests or personal relationships which have, or could be perceived to have, influenced the work reported in this article.

## Appendix Supplementary materials

Image, application 1

Image, application 2
