# Supplementary material for: Dataset on the questionnaire-based survey of sharing services users’ motivation
Source: Data Brief. 2020 Nov 5;33:106502. doi: 10.1016/j.dib.2020.106502 (PMC7680777; doi:10.1016/j.dib.2020.106502)
Supplement: Supplementary file 1 [file mmc1.docx]

Questionnaire

You are kindly invited to participate in the survey carried out by the Plekhanov Russian University of Economics to study the motivation to use shared economy services and platforms. Please fill in the questionnaire

| **Section 1** |  |  |  |  |  |  |  | |
| --- | --- | --- | --- | --- | --- | --- | --- | --- |
| Do you use shared economy platforms and services | **yes** | **no** | **Can’t say** |  |  |  |  | |
| Which ones? | **Car-sharing** | **Bike-sharing** | **Co-working** | Co-living | The housing rentals | Cloud services | None of the above | Other |
| How often do you used them | Several times per week | Several times per month | Several times per year | Once a year | Tried once or twice |  |  | |
| How old are you | 18-25 | 26-35 | 36-45 | 46-60 | over 60 |  |  | |
| Your gender | M | F |  |  |  |  |  | |
| Are you employed | Work for an employer | Self-employed | entrepreneur | Temporarily unemployed | Full time student | Not working |  | |
| **Section 2** |  |  |  |  |  |  |  | |
| Define the degree of your agreement with the following statements | **Completely agree** | **Agree** | **Inclined to agree** | **Difficult to say** | **Inclined to disagree** | **Disagree** | **Completely disagree** | |
| ***Attitude*** | **7** | **6** | **5** | **4** | **3** | **2** | **1** | |
| All things considered, I find participating in collaborative consumption to be a wise move. |  |  |  |  |  |  |  | |
| All things considered, I think participating in collaborative consumption is a good thing. |  |  |  |  |  |  |  | |
| Overall, sharing goods and services within a collaborative consumption community makes sense. |  |  |  |  |  |  |  | |
| Collaborative consumption is a better mode of consumption than selling and buying |  |  |  |  |  |  |  | |
| ***Behaviour*** | **Completely agree** | **Agree** | **Inclined to agree** | **Difficult to say** | **Inclined to disagree** | **Disagree** | **Completely disagree** | |
|  | **7** | **6** | **5** | **4** | **3** | **2** | **1** | |
| All things considered, I expect to continue collaborative consumption often in the future. |  |  |  |  |  |  |  | |
| I can see myself engaging in collaborative consumption more frequently in the future. |  |  |  |  |  |  |  | |
| It is likely that I will frequently participate in collaborative consumption communities in the future |  |  |  |  |  |  |  | |
| ***Sustainability*** | **Completely agree** | **Agree** | **Inclined to agree** | **Difficult to say** | **Inclined to disagree** | **Disagree** | **Completely disagree** | |
|  | **7** | **6** | **5** | **4** | **3** | **2** | **1** | |
| Collaborative consumption helps save natural resources. |  |  |  |  |  |  |  | |
| Collaborative consumption is a sustainable mode of consumption. |  |  |  |  |  |  |  | |
| Collaborative consumption is ecological |  |  |  |  |  |  |  | |
| Collaborative consumption is efficient in terms of using energy |  |  |  |  |  |  |  | |
| Collaborative consumption is environmentally friendly. |  |  |  |  |  |  |  | |
| ***Enjoyment*** | **Completely agree** | **Agree** | **Inclined to agree** | **Difficult to say** | **Inclined to disagree** | **Disagree** | **Completely disagree** | |
|  | **7** | **6** | **5** | **4** | **3** | **2** | **1** | |
| I think collaborative consumption is enjoyable. |  |  |  |  |  |  |  | |
| I think collaborative consumption is interesting. |  |  |  |  |  |  |  | |
| I think collaborative consumption is fun. |  |  |  |  |  |  |  | |
| I think collaborative consumption is pleasant. |  |  |  |  |  |  |  | |
| ***Reputation*** | **Completely agree** | **Agree** | **Inclined to agree** | **Difficult to say** | **Inclined to disagree** | **Disagree** | **Completely disagree** | |
|  | **7** | **6** | **5** | **4** | **3** | **2** | **1** | |
| Contributing to my collaborative consumption community improves my image within the community. |  |  |  |  |  |  |  | |
| I gain recognition from contributing to my collaborative consumption community. |  |  |  |  |  |  |  | |
| I would earn respect from others by sharing with other people in my collaborative consumption |  |  |  |  |  |  |  | |
| People in the community who contribute have more prestige than those who do not |  |  |  |  |  |  |  | |
| ***Economy*** | **Completely agree** | **Agree** | **Inclined to agree** | **Difficult to say** | **Inclined to disagree** | **Disagree** | **Completely disagree** | |
| I can save money if I participate in collaborative consumption. |  |  |  |  |  |  |  | |
| My participation in collaborative consumption benefits me financially |  |  |  |  |  |  |  | |
| My participation in collaborative consumption can improve my economic situation. |  |  |  |  |  |  |  | |
| My participation in collaborative consumption saves me time |  |  |  |  |  |  |  | |
