# Supplementary material for: Dataset on the questionnaire-based survey of sharing services users’ motivation
Source: Data Brief. 2020 Nov 5;33:106502. doi: 10.1016/j.dib.2020.106502 (PMC7680777; doi:10.1016/j.dib.2020.106502)
Supplement: Supplementary file 2 [file mmc2.docx]

**Анкета для проведения опроса о мотивах использования платформ и сервисов распределенного пользования.**

Опрос проводится онлайн среди пользователей различных платформ, приложений и сервисов совместного пользования.

| **Раздел 1** | **да** | **нет** | **Не знаю** |  |  |  |  | |
| --- | --- | --- | --- | --- | --- | --- | --- | --- |
| Используете ли вы платформы, приложения и сервисы совместного пользования (шеринга) товаров и услуг, такие как каршеринг, байк-шеринг, коворкинг, коливинг, аренда жилья, облачные сервисы и т.п. |  |  |  |  |  |  |  | |
| Какие именно? | Каршеринг | Байк-шеринг | Коворкинг | Коливинг | Аренда жилья | Облачные сервисы | Ничего из перечисленного | Другое |
| Как часто вы используете эти сервисы | Несколько раз в неделю | Несколько раз в месяц | Несколько раз в год | Раз в год | Пробовал 1-2 раза |  |  | |
| Сколько вам лет | 18-25 | 26-35 | 36-45 | 46-60 | Больше 60 |  |  | |
| Ваш пол | М | Ж |  |  |  |  |  | |
| Вы работаете | Работаю по найму | самозанятый | предприниматель | Временно не работаю | Студент очной формы | Не работаю |  | |
| **Раздел 2** |  |  |  |  |  |  |  | |
| Укажите степень согласия с указанными утверждениями | **Совершенно согласен** | **Согласен** | **Скорее согласен** | **Трудно сказать согласен или нет** | **Скорее не согласен** | **Не согласен** | **Совершенно не согласен** | |
| ***Отношение*** | **7** | **6** | **5** | **4** | **3** | **2** | **1** | |
| Я считаю, что участие в совместном потреблении полезно для общества |  |  |  |  |  |  |  | |
| Я считаю, что участие в совместном потреблении соответствует требованиям времени |  |  |  |  |  |  |  | |
| Совместное использование товаров и услуг в совместном потреблении имеет смысл |  |  |  |  |  |  |  | |
| Совместное потребление лучше, чем приобретение индивидуально товаров и услуг |  |  |  |  |  |  |  | |
| ***Поведение*** | **Совершенно согласен** | **Согласен** | **Скорее согласен** | **Трудно сказать согласен или нет** | **Скорее не согласен** | **Не согласен** | **Совершенно не согласен** | |
|  | **7** | **6** | **5** | **4** | **3** | **2** | **1** | |
| Я планирую продолжить использование совместного потребления в будущем |  |  |  |  |  |  |  | |
| Я думаю, что буду чаще использовать совместное потребление в будущем |  |  |  |  |  |  |  | |
| Я предвижу большее использование совместного потребления в будущем, если такая возможность будет |  |  |  |  |  |  |  | |
| ***Устойчивое развитие*** | **Совершенно согласен** | **Согласен** | **Скорее согласен** | **Трудно сказать согласен или нет** | **Скорее не согласен** | **Не согласен** | **Совершенно не согласен** | |
|  | **7** | **6** | **5** | **4** | **3** | **2** | **1** | |
| Совместное потребление позволяет сохранить природные ресурсы |  |  |  |  |  |  |  | |
| Совместное потребление – устойчивая модель развития |  |  |  |  |  |  |  | |
| Совместное потребление экологично |  |  |  |  |  |  |  | |
| Совместное потребление эффективно по затратам энергии |  |  |  |  |  |  |  | |
| Совместное потребление сохраняет окружающую среду |  |  |  |  |  |  |  | |
| ***Удовольствие*** | **Совершенно согласен** | **Согласен** | **Скорее согласен** | **Трудно сказать согласен или нет** | **Скорее не согласен** | **Не согласен** | **Совершенно не согласен** | |
|  | **7** | **6** | **5** | **4** | **3** | **2** | **1** | |
| Я считаю, что совместное потребление доставляет удоволетворение |  |  |  |  |  |  |  | |
| Совместное потребление – это интересно |  |  |  |  |  |  |  | |
| Совместное потребление – это весело |  |  |  |  |  |  |  | |
| Совместное потребление приятно |  |  |  |  |  |  |  | |
| ***Репутация*** | **Совершенно согласен** | **Согласен** | **Скорее согласен** | **Трудно сказать согласен или нет** | **Скорее не согласен** | **Не согласен** | **Совершенно не согласен** | |
|  | **7** | **6** | **5** | **4** | **3** | **2** | **1** | |
| Участие в совместном потреблении улучшает мой имидж в моем сообществе |  |  |  |  |  |  |  | |
| Участвуя в совместном потреблении, я получаю признание членов моего сообщества |  |  |  |  |  |  |  | |
| Участвуя в совместном потреблении, я завоевываю уважение членов моего сообщества |  |  |  |  |  |  |  | |
| Те, кто участвует в совместном потреблении, имеют больший авторитет, чем те, кто не участвует |  |  |  |  |  |  |  | |
| ***Экономия*** | **Совершенно согласен** | **Согласен** | **Скорее согласен** | **Трудно сказать согласен или нет** | **Скорее не согласен** | **Не согласен** | **Совершенно не согласен** | |
|  |  |  |  |  |  |  |  | |
| Я могу сэкономить, используя совместное потребление |  |  |  |  |  |  |  | |
| Мое участие в совместном потреблении выгодно мне в финансовом плане |  |  |  |  |  |  |  | |
| Мое участие в совместном потреблении может улучшить мое экономическое положение |  |  |  |  |  |  |  | |
| Участие в совместном потреблении экономит мне время |  |  |  |  |  |  |  | |
